# Bird biodiversity assessments in temperate forest: the value of point count versus acoustic monitoring protocols

**DOI:** 10.7717/peerj.973

**Published:** 2015-05-19

**Authors:** Brian T. Klingbeil, Michael R. Willig

**Affiliations:** Department of Ecology & Evolutionary Biology and Center for Environmental Sciences & Engineering, University of Connecticut, Storrs, CT, USA

**Keywords:** ARU, Avian, Conservation, Diversity, Interior forest, Long-term monitoring, Management, Methodology

## Abstract

Effective monitoring programs for biodiversity are needed to assess trends in biodiversity and evaluate the consequences of management. This is particularly true for birds and faunas that occupy interior forest and other areas of low human population density, as these are frequently under-sampled compared to other habitats. For birds, Autonomous Recording Units (ARUs) have been proposed as a supplement or alternative to point counts made by human observers to enhance monitoring efforts. We employed two strategies (i.e., simultaneous-collection and same-season) to compare point count and ARU methods for quantifying species richness and composition of birds in temperate interior forests. The simultaneous-collection strategy compares surveys by ARUs and point counts, with methods matched in time, location, and survey duration such that the person and machine simultaneously collect data. The same-season strategy compares surveys from ARUs and point counts conducted at the same locations throughout the breeding season, but methods differ in the number, duration, and frequency of surveys. This second strategy more closely follows the ways in which monitoring programs are likely to be implemented. Site-specific estimates of richness (but not species composition) differed between methods; however, the nature of the relationship was dependent on the assessment strategy. Estimates of richness from point counts were greater than estimates from ARUs in the simultaneous-collection strategy. Woodpeckers in particular, were less frequently identified from ARUs than point counts with this strategy. Conversely, estimates of richness were lower from point counts than ARUs in the same-season strategy. Moreover, in the same-season strategy, ARUs detected the occurrence of passerines at a higher frequency than did point counts. Differences between ARU and point count methods were only detected in site-level comparisons. Importantly, both methods provide similar estimates of species richness and composition for the region. Consequently, if single visits to sites or short-term monitoring are the goal, point counts will likely perform better than ARUs, especially if species are rare or vocalize infrequently. However, if seasonal or annual monitoring of sites is the goal, ARUs offer a viable alternative to standard point-count methods, especially in the context of large-scale or long-term monitoring of temperate forest birds.

## Introduction

Standardized long-term programs for monitoring biodiversity that span large geographic areas are needed to determine species responses to global change and to inform conservation efforts. Effective monitoring programs identify changes in species distributions, assess population trends and evaluate the efficacy of management practices. In this context, birds represent one of the most well studied groups of wildlife, with a history of long-term studies, including a number of large-scale monitoring programs (e.g., Christmas Birds Count, North American Breeding Bird Survey). Nonetheless, considerable gaps exist in our knowledge of the current status and recent population trends of forest birds (Sauer, Fallon & Johnson, 2003; Blancher et al., 2009; Francis, Blancher & Phoenix, 2009).

Point-count surveys, where an observer records all birds seen or heard at a point location for a specified time (Ralph, Sauer & Droege, 1995), are the most common survey method for long-term avian studies (Rosenstock et al., 2002). Interior forest and other areas of low human population density are frequently under-sampled in such large-scale monitoring programs because surveys are often conducted by volunteers (Francis, Blancher & Phoenix, 2009). Surveys by volunteers are often employed because they are a cost-effective method, and the involvement of non-scientists in science (i.e., citizen science) enhances public appreciation of biodiversity and conservation (Dickinson et al., 2012; Price & Lee, 2013). Furthermore, with freely accessible and up-to-date survey results (e.g., eBird) as well as digital tools that enable identification of species (e.g., Merlin bird ID and xeno-canto) and changes in distributions, citizen science initiatives have the potential to address critical needs in science and conservation. Nonetheless, volunteer-based surveys are not without drawbacks, including data quality concerns (e.g., variation in identification accuracy related to age, education, collection skills, and length of participation in the program; Dickinson, Zuckerberg & Bonter, 2010). The use of Autonomous Recording Units (ARUs) to survey birds and other taxa has been suggested as a supplement to enhance monitoring efforts, especially in remote or inaccessible areas, like interior forest Haselmayer & Quinn, 2000; Hobson et al., 2002; Acevedo & Villanueva-Rivera, 2006; Hutto & Stutzman, 2009; Campbell & Francis, 2011; Venier et al., 2012; Tegeler, Morrison & Szewczak, 2012; Furnas & Callas, 2015.

ARUs, reduce several types of bias associated with point-count surveys and facilitate consistent data collection among surveys and sites that improve detection of species and estimation of species richness. By using ARUs, biases and problems associated with monitoring can be reduced because: (1) data collection does not depend on observer skill level, reducing observer bias; (2) recorders can be left unattended to regularly record vocalizations for long periods of time, reducing temporal restrictions (Hobson et al., 2002; Tegeler, Morrison & Szewczak, 2012); (3) multiple sites can be monitored simultaneously, eliminating temporal bias (Venier et al., 2012; Tegeler, Morrison & Szewczak, 2012); (4) data collection provides permanent records of vocalizations that can be played repeatedly and, if necessary, independently verified by multiple experts, reducing identification errors (Rempel et al., 2005); and (5) human observers are absent during recordings, eliminating attractions or deterrents for some bird species, reducing biases in detectability (Bye, Robel & Kemp, 2001). Furthermore, ARUs have the potential to significantly reduce the number of trained observers that need to be sent to the field, freeing time and personnel resources during field seasons that could be spent surveying for species undetectable by acoustic approaches or accomplishing other scientific or management goals.

Like any method, ARUs suffer from a number of shortcomings. They are subject to malfunction or breakage, and their performance may be affected by adverse environmental conditions for extended periods of time (e.g., microphones can become waterlogged reducing sound quality). Most importantly, ARUs lack the visual component of traditional point count surveys, making detection more difficult for vocally cryptic species and reducing reliability of estimates for species abundance. As a result, ARUs are often suggested as a supplement to point counts, but have not been embraced as a viable alternative to be used in place of them (e.g., Venier et al., 2012; Tegeler, Morrison & Szewczak, 2012; Furnas & Callas, 2015).

Most previous comparisons between point counts and ARUs have generally relied on assessments when point counts conducted by a trained observer and audio recordings made by a single ARU are paired in time and space (e.g., Haselmayer & Quinn, 2000; Hobson et al., 2002; Acevedo & Villanueva-Rivera, 2006; Celis-Murillo, Deppe & Allen, 2009; Hutto & Stutzman, 2009; but see Tegeler, Morrison & Szewczak, 2012), although additional studies have made comparisons between an observer and multiple ARU models to evaluate differences between equipment types (Venier et al., 2012; Rempel et al., 2013). Such studies are important for evaluating new technologies and provide information to conservation managers in a rapidly developing field (with many new equipment options). However, these studies may not provide the best assessment of ARUs as a monitoring alternative because comparisons fail to capitalize on one of ARUs primary assets: repeated unattended surveys over an extended time period. Consequently, we use two assessment strategies (Table 1) to identify if differences exist in the efficacy of point count and ARU methods with respect to estimating species richness and composition of bird communities in interior forest.

**Table 1:** Methodological details for comparisons of point count and ARU methods. Details of two assessment strategies used to compare point count and ARU methods for estimating richness and composition of temperate interior forest bird communities.

Method details	Simultaneous-collection		Same-season	
	ARU	Point count	ARU	Point count
Surveys per site	3	3	50	10
Survey duration	10 min	10 min	2 min	10 min
Total number of surveys	60	60	1,000	200
Survey effort	600 min	600 min	2,000 min	2,000 min

The simultaneous-collection strategy compares surveys by ARUs and point counts, for which methods are exactly matched in time, location, and survey duration. The simultaneous-collection strategy is similar to previous studies that compare point counts and ARUs in that an observer stands next to an ARU, and both simultaneously collect data. Consequently, results from this strategy can be compared to previous studies to determine if the performance of ARUs in temperate interior forest is similar to other habitats (e.g., burned conifer forest-riparian gradient-Hutto & Stutzman, 2009; boreal forest-Venier et al., 2012; alpine meadows-Tegeler, Morrison & Szewczak, 2012; BBS survey route-Rempel et al., 2013). The same-season strategy compares surveys from ARUs and point counts conducted at the same locations throughout the breeding season, but methods differ in the number, duration, and frequency of surveys (but total sample effort is equal). This comparison evaluates if a substantially higher number of days sampled by ARUs corresponds to different estimates of species richness and composition than do point counts, without confounding estimates with the effects of increased effort. Holding total sample effort (i.e., number of survey minutes) constant between methods represents a conservative estimate of the utility of ARUs, because they can record for extended time (hours per day and number of days) without additional effort or cost.

## Methods

**Figure 1:**
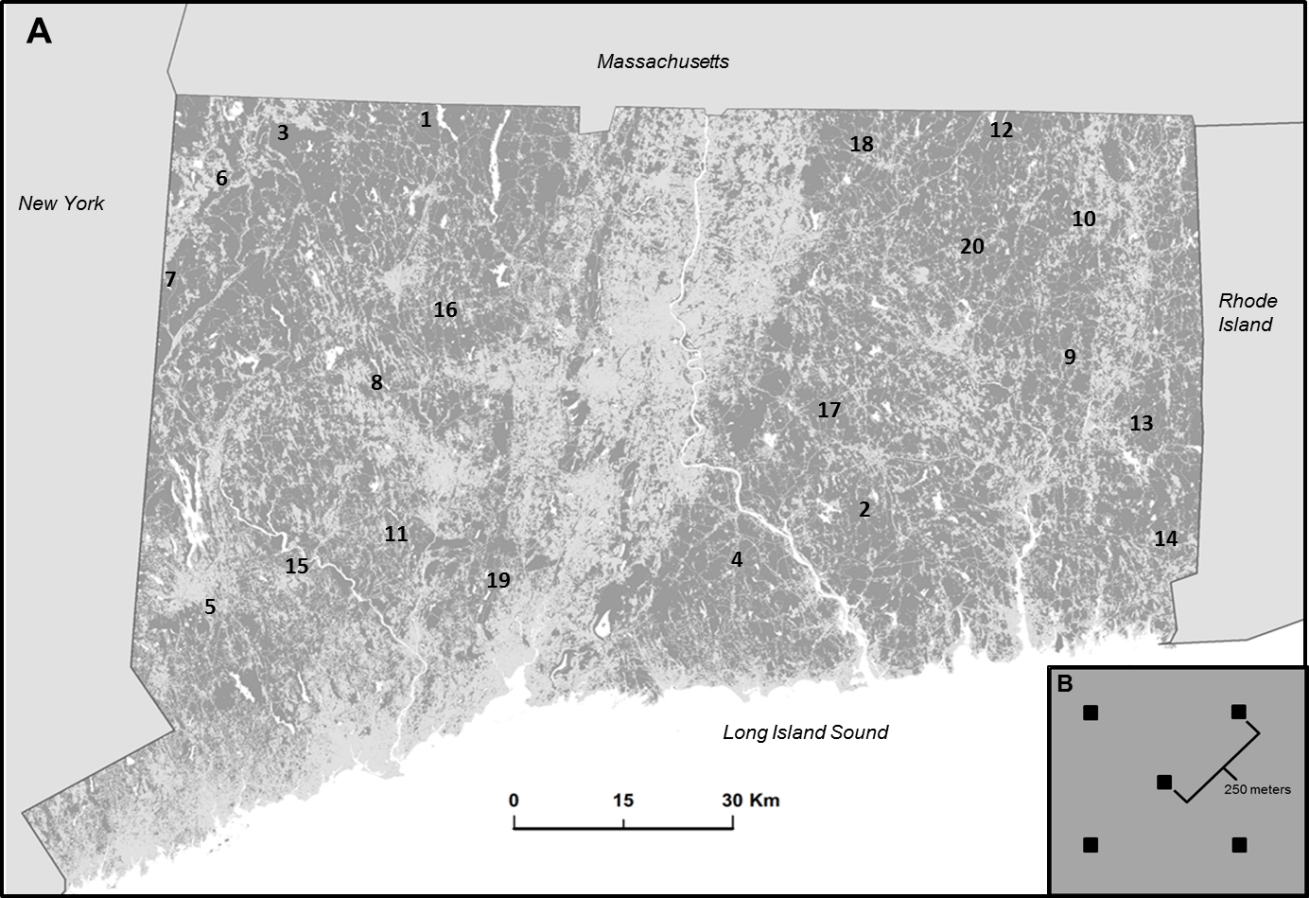
Study location and design. (A) Map of study area in Connecticut, USA represented by forest (dark gray) non forest (light gray) and water (white). Location of 20 interior forest sites are indicated by number (see Supplemental Information for geographic coordinates). (B) Diagram illustrating the arrangement of five plots (black squares) within a site. Each square represents a paired ARU and point count location.

Research was conducted in eastern deciduous and coniferous forests of Connecticut, USA, excluding coastal areas. Currently, 60% of land cover in the state is forest, dominated by oak-hickory and northern hardwood forest types, although pine forests are common along the northern border of the state (Butler, 2013). Like many areas, Connecticut has experienced profound anthropogenic alteration of landscapes (Drummond & Loveland, 2010), so that forest currently exists as patches or fragmented parcels of various sizes and ages, interwoven with various types of human-altered land covers (i.e., urban and suburban developments, agricultural fields, road networks, and power line rights of way; Fig. 1A).

Twenty sites were established on public land within interior forest patches of various sizes and shapes. Roughly one-third of the forests in the lower 48 states are on public lands, supporting 45% of the US distribution of 149 obligate forest bird species, and represent the largest unfragmented forests in many regions (NABCI, 2011). Site locations were selected by processing a 2010 land cover map (CLEAR, 2010b) with the Landscape Fragmentation Tool (LFT v2.0; CLEAR, 2010a) add-on to Arc Toolbox to identify suitable sites with sufficient area of interior forest (i.e., forest pixels located at least 100 m from non-forest pixels) to contain 5 plots, each containing an ARU. Within each site, plots were spaced at least 250 m apart to avoid overlap in the sampled acoustic environment (Fig. 1B). Sites were at least 10 km from other sites and from Long Island Sound. Prospective sites were visited and ground-truthed to evaluate accuracy of land cover maps and to ensure accessibility.

Birds were surveyed via both point counts (Ralph, Sauer & Droege, 1995) and recordings from ARUs. For point counts, each plot within each site (Fig. 1B) was visited on two occasions during the breeding season (May 21–August 1, 2012) and sampled with a 10 min survey. Because of logistic and weather constraints, the order in which sites were visited was not fully randomized. However, all sites were visited once before any received a second survey, and the order of site visits differed between the two occasions. Surveys were conducted within the first 4 h of local sunrise, and all species heard or seen were recorded as present. In addition to point counts, each of the 5 plots within a site contained an ARU (Wildlife Acoustics Song Meter Sm2 +) and was surveyed for 4 h on a daily basis, beginning at local sunrise during the same time period as point counts. ARUs were equipped with two omni-directional microphones (flat frequency response between 20 Hz and 20 kHz) and signals were sampled at 24,000 Hz. ARUs with microphones pointing horizontally were attached to trees at a height of 2 m and were located within 3 m of the point count location. Recordings were analyzed and spectrograms were viewed with Song Scope software (Wildlife Acoustics Inc., Maynard, Massachusetts, USA). To assist in identification of species, field recordings were compared by listening to recordings and viewing sonograms of previously identified species obtained from the Macaulay Library at the Cornell Lab of Ornithology. We focus our analyses on two orders (i.e., Passeriformes and Piciformes) that are well represented and comprise the majority of species in temperate interior forest (Monkkonen, 1994; Keddy & Drummond, 1996). We followed the nomenclature and taxonomic recommendations of the North American Classification Committee of the American Ornithologists’ Union (Chesser et al., 2013).

Two assessment strategies were used to compare forest bird richness and composition between ARU and point count methods (Table 1). In the same-season strategy, point count data were compared to a random subset of recordings collected throughout the breeding season. For each site, recordings from 5 ARUs were sampled by randomly selecting a plot and a 2-minute time period separately on each of 50 days during the breeding season, exclusive of the 2 days when point counts were conducted at particular sites (to eliminate biases associated with observer presence). This approach results in equal sample effort in recordings and point count surveys for each site (Table 1). This acoustic sampling strategy represents a compromise between maximizing the number of days sampled, while including a sufficient amount of time per day to capture multiple vocalizations of a species.

In the simultaneous-collection strategy, we evaluate if the same species are identified by point counts and ARUs when paired in time, location, duration, and observer (Table 1). Three plots from each site were selected randomly and a 10 min recording that corresponded to a 10 min point count conducted by the same observer was selected (i.e., 60 samples from each method paired in time, location, and observer). Plots within sites were randomly selected when possible but some sites did not have more than three paired recordings because not all ARUs were recording at the time of point counts due to weather, animal induced damage, or equipment malfunction.

To determine if the local environment influences the efficacy of methods, the habitat surrounding ARUs and point count locations was quantified. At each plot, five habitat characteristics were estimated. Elevation was determined with a handheld GPS receiver. Slope was estimated on a scale of 0–3, with 0 indicating no slope and 3 indicating a very steep slope (>45°). Canopy openness was estimated with a concave spherical densiometer at the center of a plot and at a distance of 5 m in each of the 4 cardinal directions. Understory density was estimated on a scale from 0 to 5, with 0 indicative of completely open understory commonly associated with old growth coniferous forest and with 5 indicative of very dense understory that is commonly associated with dense patches of mountain laurel (*Kalmia latifolia*) or invasive Japanese barberry (*Berberis thunbergii*). Ground cover of leaves (including pine needles) and herbs was visually estimated as the percent area covered within a 5 m radius circle at the center of each plot.

We evaluated if differences in species richness or in species composition exist between ARU and point count methods, and determine if differences arise as a consequence of assessment strategy. We held total survey effort (i.e., number of minutes) constant in comparisons of data between methods for each strategy. We used paired *t*-tests to assess if differences in estimates of richness exist between approaches in the same-season strategy. For comparisons based on the simultaneous-collection strategy, we partitioned site richness (gamma) into within (alpha) and among (beta) plot richness. Alpha (*α*) is the average richness of plots within a site. Beta (*β*) is the average number of compartments (i.e., groups of plots with similar species composition that are distinct from other such groups of plots) and reflects the heterogeneity of a site (from the perspective of the sampled birds). Gamma (*γ*) is the cumulative richness of a site (pooling all three plots). We used a multiplicative approach (*αβ* = *γ*) to determine partitions (Whittaker, 1972). Paired *t*-tests quantified differences in richness between methods at each of these 3 levels.

The frequency of occurrence of each species was used to characterize species composition of the region (interior forest of Connecticut) separately for each combination of method and strategy. Frequency of occurrence in the simultaneous-collection strategy was determined by counting the number of times a species was observed at plots (*n* = 60) via ARUs or point counts. Frequency of occurrence in the same-season strategy was determined by counting the number of times a species was observed at sites (*n* = 20) via ARUs or point counts. Estimates of regional species composition derived from point counts and ARUs were compared with chi-square randomization tests separately for each strategy. In addition, we evaluated if a taxonomic bias existed between methods by comparing the frequency of occurrence of birds in the orders Piciformes and Passeriformes separately with paired *t*-tests for each strategy.

Lastly, we determined if differences in species composition between point counts and ARUs were related to habitat characteristics of forest interior plots. We used the additive inverse of Jaccard’s similarity coefficient (J) to estimate dissimilarity in species composition between methods in the simultaneous collection strategy. Spearman rank correlations evaluated associations between habitat characteristics and species dissimilarity.

## Results

Sites were characterized by low canopy openness, low understory density, and a greater percentage of leaf and needle coverage than of herbaceous cover. Mean elevation ranged from 105 to 375 m above sea level (Table S1). Forty-one species were identified with point counts and thirty-nine species were identified with ARUs (Table 2). Five species (Canada Warbler, Great Crested Flycatcher, Hooded Warbler, Yellow-bellied Sapsucker, and Yellow-throated Vireo) were identified only with point counts, whereas three species (Common Raven, Gray Catbird, and Winter Wren) were identified only with ARUs.

**Table 2:** Frequency of occurrence of birds in temperate interior forest identified with two assessment strategies (i.e., simultaneous-collection and same-season). Methodological details of each assessment strategy are listed in Table 1 and described in the text. A dash indicates the species was not identified with a particular strategy.

Order	Family	Scientific name	Common name	Simultaneous-collection		Same-season	
				Point count	ARU	Point count	ARU
Piciformes	Picidae	*Melanerpes erythrocephalus*	Red-headed Woodpecker	0.02	0.00	0.10	0.05
Piciformes	Picidae	*Melanerpes carolinus*	Red-bellied Woodpecker	0.07	0.07	0.45	0.40
Piciformes	Picidae	*Sphyrapicus varius*	Yellow-bellied Sapsucker	0.02	0.00	0.05	0.00
Piciformes	Picidae	*Picoides pubescens*	Downy Woodpecker	0.02	0.00	0.25	0.50
Piciformes	Picidae	*Picoides villosus*	Hairy Woodpecker	0.05	0.03	0.55	0.40
Piciformes	Picidae	*Colaptes auratus*	Northern Flicker	0.02	0.00	0.40	0.15
Piciformes	Picidae	*Dryocopus pileatus*	Pileated Woodpecker	0.02	0.00	0.15	0.30
Passeriformes	Tyrannidae	*Contopus virens*	Eastern Wood-pewee	0.38	0.28	0.90	0.95
Passeriformes	Tyrannidae	*Sayornis phoebe*	Eastern Phoebe	0.02	0.02	0.15	0.05
Passeriformes	Tyrannidae	*Myiarchus crinitus*	Great Crested Flycatcher	–	–	0.05	0.00
Passeriformes	Vireonidae	*Vireo flavifrons*	Yellow-throated Vireo	–	–	0.25	0.00
Passeriformes	Vireonidae	*Vireo olivaceus*	Red-eyed Vireo	0.55	0.68	1.00	1.00
Passeriformes	Corvidae	*Cyanocitta cristata*	Blue Jay	0.25	0.23	0.85	0.85
Passeriformes	Corvidae	*Corvus brachyrhynchos*	American Crow	0.03	0.07	0.40	0.50
Passeriformes	Corvidae	*Corvus corax*	Common Raven	–	–	0.00	0.05
Passeriformes	Paridae	*Baeolophus bicolor*	Tufted Titmouse	0.27	0.30	0.75	0.95
Passeriformes	Paridae	*Poecile atricapillus*	Black-capped Chickadee	0.13	0.17	0.90	0.90
Passeriformes	Sittidae	*Sitta canadensis*	Red-breasted Nuthatch	0.03	0.02	0.15	0.05
Passeriformes	Sittidae	*Sitta carolinensis*	White-breasted Nuthatch	0.13	0.17	0.90	0.95
Passeriformes	Troglodytidae	*Troglodytes heimalis*	Winter Wren	–	–	0.00	0.25
Passeriformes	Turdidae	*Catharus fuscescens*	Veery	0.30	0.27	0.80	0.85
Passeriformes	Turdidae	*Catharus guttatus*	Hermit Thrush	0.13	0.13	0.65	0.55
Passeriformes	Turdidae	*Hylocichla mustelina*	Wood Thrush	0.25	0.27	0.80	0.90
Passeriformes	Turdidae	*Turdus migratorius*	American Robin	0.02	0.02	0.05	0.10
Passeriformes	Mimidae	*Dumetella carolinensis*	Gray Catbird	–	–	0.00	0.05
Passeriformes	Parulidae	*Seiurus aurocapilla*	Ovenbird	0.85	0.83	1.00	1.00
Passeriformes	Parulidae	*Parkesia motacilla*	Louisiana Waterthrush	0.02	0.02	0.05	0.00
Passeriformes	Parulidae	*Parkesia noveboracensis*	Northern Waterthrush	–	–	0.05	0.05
Passeriformes	Parulidae	*Mniotilta varia*	Black-and-white Warbler	0.05	0.07	0.20	0.60
Passeriformes	Parulidae	*Geothlypis trichas*	Common Yellowthroat	0.00	0.02	0.05	0.10
Passeriformes	Parulidae	*Setophaga citrina*	Hooded Warbler	–	–	0.05	0.00
Passeriformes	Parulidae	*Setophaga ruticilla*	American Redstart	0.05	0.03	0.05	0.60
Passeriformes	Parulidae	*Setophaga cerulea*	Cerulean Warbler	0.02	0.02	0.05	0.20
Passeriformes	Parulidae	*Setophaga magnolia*	Magnolia Warbler	–	–	0.05	0.15
Passeriformes	Parulidae	*Setophaga caerulescens*	Black-throated Blue Warbler	0.03	0.02	0.15	0.10
Passeriformes	Parulidae	*Setophaga pinus*	Pine Warbler	0.03	0.02	0.25	0.25
Passeriformes	Parulidae	*Setophaga virens*	Black-throated Green Warbler	0.12	0.10	0.20	0.55
Passeriformes	Parulidae	*Cardellina canadensis*	Canada Warbler	0.02	0.00	0.05	0.00
Passeriformes	Emberizidae	*Pipilo erythrophthalmus*	Eastern Towhee	0.03	0.05	0.15	0.30
Passeriformes	Emberizidae	*Spizella passerina*	Chipping Sparrow	0.05	0.02	0.25	0.45
Passeriformes	Cardinalidae	*Piranga olivacea*	Scarlet Tanager	0.30	0.33	0.85	1.00
Passeriformes	Cardinalidae	*Cardinalis cardinalis*	Northern Cardinal	–	–	0.10	0.20
Passeriformes	Cardinalidae	*Pheucticus ludovicianus*	Rose-breasted Grosbeak	–	–	0.15	0.20

### Simultaneous-collection strategy

Alpha or beta components of richness (Table 3) were not significantly different between methods (Table 4). In contrast, gamma was higher for point counts than ARUs. Regional species composition did not differ between survey types (X^2^ = 13.11, *p* = 1.0). However, a significant difference existed between methods in the frequency of occurrence of birds in Piciformes (Table 5). Dissimilarity (1-J) of species identified by surveys and recordings varied from 0.0 to 0.5 (Table 3), and was associated negatively with elevation (rho = − 0.511, *p* = 0.021; Table 6).

**Table 3:** Comparison of methods based on simultaneous-collection strategy. Estimates of richness and dissimilarity from point count and ARU methods based on data from the simultaneous-collection strategy. Richness is partitioned into alpha, beta, and gamma components based on the multiplicative model (Whittaker, 1972). Alpha refers to the mean richness of 3 plots within each site. Gamma refers to the cumulative richness of 3 plots within each site. Beta is the average number of compartments in a site and reflects the heterogeneity of a site. Dissimilarity (1 − Jaccard’s coefficient) estimates the difference in species composition for each site determined by point count versus ARU methods. Total number of species identified by ARU and point count methods with the simultaneous-collection strategy is indicated by first number in parentheses after each site name. The second number in parentheses refers to richness estimated with ARU and point count methods with the same-season strategy (See Table 1 for differences in effort between strategies).

Site number	Site	Alpha		Beta		Gamma		Dissimilarity
		Point count	ARU	Point count	ARU	Point count	ARU	
1	Algonquin (8, 20)	3.67	3.67	1.91	1.91	7	7	0.25
2	Babcock (5, 17)	3.00	3.00	1.67	1.33	5	4	0.20
3	Canaan (10, 20)	3.67	4.00	2.18	2.00	8	8	0.40
4	Cockaponsett (9, 23)	4.33	3.67	1.85	1.91	8	7	0.33
5	Collis (15, 23)	5.67	5.33	2.29	2.25	13	12	0.33
6	Housatonic (9, 14)	4.67	4.67	1.93	1.71	9	8	0.11
7	Macedonia (7, 19)	3.33	3.33	1.80	1.80	6	6	0.29
8	Mattatuck (13, 28)	7.67	7.33	1.70	1.50	13	11	0.15
9	Mohegan (9, 21)	4.67	4.33	1.50	1.85	7	8	0.33
10	Natchaug (8, 16)	4.67	4.67	1.71	1.50	8	7	0.13
11	Naugatuck (7, 16)	3.67	2.67	1.91	2.25	7	6	0.14
12	Nipmuck (10, 21)	4.67	4.67	2.14	1.93	10	9	0.10
13	PachaugN (8, 15)	3.33	3.67	2.40	2.18	8	8	0.00
14	PachaugS (9, 18)	4.33	2.33	1.85	2.57	8	6	0.44
15	Paugusset (8, 17)	2.67	3.33	2.25	1.80	6	6	0.50
16	Roraback (10, 23)	4.00	4.00	2.25	2.25	9	9	0.20
17	Salmon (14, 23)	4.67	6.00	2.36	1.83	11	11	0.43
18	Shenipsit (9, 17)	4.33	4.00	2.08	1.75	9	7	0.22
19	Sleeping (9, 16)	5.00	5.33	1.60	1.50	8	8	0.22
20	UConn (10, 25)	3.33	4.67	2.10	1.93	7	9	0.40

**Table 4:** Differences in estimates of richness from the simultaneous-collection strategy. Results from two-tailed significance tests (paired *t*-test) to evaluate mean differences in richness components estimated from the simultaneous-collection strategy. Significant relationships are indicated in bold.

Component	Point count	ARU	*t*-statistic	df	*p*-value
Alpha	4.27	4.23	0.204	19	0.841
Beta	1.97	1.89	1.315	19	0.204
Gamma	8.35	7.85	2.236	19	**0.038**

**Table 5:** Differences in frequency of occurrence for simultaneous-collection and same-season strategies. Results from two-tailed significance tests (paired *t*-test) to evaluate mean differences in frequency of occurrence of birds from two orders identified with point count and ARU methods. Comparisons were made separately for each assessment strategy. Significant relationships are indicated in bold.

Strategy	Order	n	Point count	ARU	*t*-statistic	df	*p*-value
Simultaneous-collection							
	Piciformes	7	0.029	0.014	6.000	6	**<0.001**
	Passeriformes	26	0.156	0.159	−0.498	25	0.623
Same-season							
	Piciformes	7	0.279	0.257	0.333	6	0.751
	Passeriformes	36	0.342	0.408	−2.646	35	**0.012**

**Table 6:** Relationships between site characteristics and dissimilarity. Spearman Rank correlations (Rho) and associated *p*-values between habitat characteristics and Jaccard’s dissimilarity coefficient. For each site Jaccard’s Index evaluates differences in species composition identified with Point count and ARU methods determined with the simultaneous collection strategy. Significant relationships are indicated in bold.

Habitat characteristic	Rho	*p*-value
Elevation	−0.511	**0.021**
Slope	−0.175	0.462
Understory density	0.308	0.187
Canopy openness	0.147	0.537
Ground cover	−0.327	0.159
Herb cover	0.057	0.811

### Same-season strategy

Compared to point counts, ARUs result in greater estimates of richness at sites (Paired *t*-test: *t* = − 2.7979, *p* = 0.012). Nevertheless, point count and ARU methods resulted in similar estimates of species richness in the region (i.e. , 38 species by ARUs and 40 species by point counts). Species composition was similar between methods (X^2^ = 46.26, *p* = 0.999). Although both methods produced similar estimates of regional species composition, at the site level, passerines were more frequently detected by ARUs than by point counts (Table 5).

## Discussion

In general, ARU and point count methods provided similar estimates of species composition for the region and similar estimates of richness for individual plots within sites. Conversely, methods differed in estimates of richness at the site level and relationships were dependent on assessment strategy. Comparison of results between assessment strategies provides insight into why other studies have found that ARUs can produce lower, similar, or higher estimates of species richness compared to point counts (e.g., Haselmayer & Quinn, 2000; Hobson et al., 2002; Acevedo & Villanueva-Rivera, 2006; Celis-Murillo, Deppe & Allen, 2009; Hutto & Stutzman, 2009).

### Same-season strategy

ARUs offer a viable alternative to standard point-count methods, especially in the context of large-scale or long-term avian species richness surveys of temperate forest birds. We found no difference in species composition of the regional community detected by point counts or ARUs. Furthermore, even when sample effort was held constant between protocols (representing a conservative estimate of a potential ARU sampling protocol) ARUs identified a greater number of species at sites than point count surveys. This is likely because each site was sampled on 50 different days with ARUs rather than only 2 different days with point counts. This is a clear advantage of ARU methods. Repeated visits to sites over the course of the breeding season should sample the same community of birds because the majority of forest bird species are territorial and breeding is relatively synchronous. Consequently, it is likely that higher richness estimates based on additional surveys with ARUs represent improved estimates, rather than changes in space use by species.

Passerines were more frequently identified by ARUs than by point counts in the same-season strategy. This may reflect temporal constraints associated with traditional point count surveys. The optimal period for detecting species is when they are most vocal, usually when they are establishing and defending breeding territories (Anderson, Ohmart & Rice, 1981; Best, 1981; Ralph, 1981; Skirven, 1981). Hence, typical point count surveys of breeding birds in this region begin in mid-May and end in July. However, three problems may arise with this standard protocol. First, some non-migratory or short-distance migratory species may be missed or underestimated by surveys that target migratory species during such a narrow temporal window. This is possible because some residents or short-distant migrants establish territories and breed before long distance migrants arrive, hence vocalizations may have significantly decreased by the time traditional surveys begin (Hejl & Thompson, 2000). Second, if a small number of observers are tasked with conducting point counts for a region, sites will rarely be sampled more than a few times in the period when migrants are most vocal, and weeks may pass between visits to sites, potentially missing the most vocal periods for some species at some sites. This problem is only exacerbated if monitoring programs increase in geographic area or numbers of trained observers are reduced because of budget constraints. Third, as effects of climate change become more pronounced, regional variation in arrival times of migrants may increase, with some species arriving earlier and others delaying migration (Walther et al., 2002; Jenni & Kery, 2003; Van Buskirk, Mulvihill & Leberman, 2009), further complicating the planning of point count surveys. ARUs do not suffer from the same constraints as point counts, since they can be placed at multiple sites to record simultaneously for extended periods. Furthermore, if ARUs are in place well before migrants historically arrive, they will be able to capture vocalizations from residents that may breed earlier in the season, and they can be used to identify if particular species are returning from their wintering grounds earlier in the season in response to changes in climate and altered phenology.

### Simultaneous-collection strategy

Fewer species were identified from ARUs than from point counts when data were collected simultaneously. A potential explanation for this difference reflects a common criticism of ARUs: they do not allow visual cues (except for spectrograms) to aide in species identification, representing a shortcoming of audio recording devices. Woodpeckers (Piciformes) in particular, were less frequently identified from ARUs than from point counts. Compared to other groups of birds, little research has been conducted on acoustic communication in woodpeckers (Stark, Dodenhoff & Johnson, 1998). The functions of the majority of acoustic signals used by woodpeckers are not fully understood, and variation in their acoustic behavior has received little attention (Tremain, Swiston & Mennill, 2008). Woodpeckers typically have larger territories and vocalize less frequently compared to most song birds (Blackburn, Lawton & Gaston, 1998; Farnsworth et al., 2002). Moreover, it is unknown if the presence of an observer affects the frequency of acoustic signals by these birds (i.e., warning calls or drumming). Only songs and calls were used to identify bird species from ARU recordings, so even if drumming was recorded (which it frequently was) it was not used as the only source of information for identification. Use of drumming was not used for identification in the field either, however drumming could be used to direct an observer’s attention to facilitate visual identification of the birds, even if the individual was not otherwise vocalizing. This increases the likelihood of detection and could represent a bias in species detection frequencies between methods for woodpeckers. Indeed, when comparing single-visit recordings with field observations, the latter are likely more effective at identifying rarely heard species, whereas recordings would be more beneficial in areas of high species richness when many birds are calling, and repeated listening and viewing of spectrograms can be employed to identify species with overlapping vocalizations (Haselmayer & Quinn, 2000; Hutto & Stutzman, 2009). Importantly, when data collected from ARUs and point counts were compared for the full season (i.e., same-season strategy), no significant difference existed in the frequency of occurrence of woodpeckers. Ultimately, the advantage of being able to sample more frequently or over a longer time frame with ARUs may offset the lack of visual detection associated with ARUs, making ARUs a viable solution to detecting species that vocalize less frequently.

Differences in the number of species detected between paired point counts and ARUs may also reflect variation among sites within which surveys were conducted. We found no differences in estimates of alpha or beta diversity between surveys and recordings, but we did identify significant difference between estimates of gamma diversity. This intimates that recordings and surveys were equally efficient in capturing variation in richness that manifests at the plot level and to account for microhabitat variation within sites. Conversely, variation among sites had the greatest influence on the ability of recordings to estimate richness when compared with field observations. This is critically important to consider from a monitoring perspective, because it suggests that differences between field observations and recordings may be habitat-specific, and that ARUs may not perform equally in all environments. Accordingly, if study designs incorporate multiple habitat types, preliminary analyses should be conducted to determine if biases exist between habitats included in the program.

Dissimilarity in the identity of species between field surveys and recordings was negatively related to elevation, indicating that lower elevation sites generally shared the lowest proportion of species between surveys and recordings. However, mean elevation of plots only ranged from 96.5–389.33 m above sea level, thus it is unlikely that changes in environmental characteristics (i.e., temperature, solar irradiation, precipitation, productivity, or habitat type) often associated with changes in elevation affected these patterns. Other general site characteristics (e.g., canopy openness or understory density) that might be expected to play a role in interfering with the audio or visual components of surveys were unrelated to differences between recordings and field observations, suggesting that unmeasured aspects of forest structure that co-vary with elevation in this system may influence bird identification (e.g., diversity or richness of trees, vertical heterogeneity of forests). Alternatively, as evidenced by fewer detections of woodpeckers with ARU methods in the simultaneous-collection strategy, not all species have equal detection probabilities. Consequently, it is possible that sites at lower elevations contain a greater number of species with lower acoustic detection probabilities as a result of species-specific elevational associations.

## Conclusions

ARUs provide data on the presence of birds that are comparable to that obtained by field observers. Our results support previous studies in other habitats (Haselmayer & Quinn, 2000; Hobson et al., 2002; Campbell & Francis, 2011; Tegeler, Morrison & Szewczak, 2012) in suggesting that ARUs can be used as a viable alternative to skilled field observers to collect data. However, the full benefit of ARUs will only be realized when they are deployed for an extended duration, rather than a single visit to sites. If single visits to sites or short-term monitoring are the goal, point counts will likely perform better than ARUs, especially if species are rare, or vocalize infrequently. Conversely, if long-term or large-scale monitoring programs are to provide useful estimates to facilitate adaptive management in the face of changing climate and habitats, efforts need to be made to reduce biases and constraints associated with traditional sampling approaches. ARUs do not suffer from the same constraints or biases as do point counts (although they do suffer from different biases). When surveys are executed across remote or large geographic areas, use of ARUs can be logistically and financially more efficient than point counts, creating a permanent record that can easily be archived and shared, and represent important tools for use by biodiversity scientists, conservation biologists or land managers.

## Supplemental Information

10.7717/peerj.973/supp-1 Supplemental Information 1 Supplementary material

## Additional Information and Declarations

### Field Study Permissions

The following information was supplied relating to field study approvals (i.e., approving body and any reference numbers): State of Connecticut Department of Environmental Protection, permit number 1113002.
