# Supplementary material for: Bird biodiversity assessments in temperate forest: the value of point count versus acoustic monitoring protocols
Source: PeerJ. 2015 May 19;3:e973. doi: 10.7717/peerj.973 (PMC4451018; doi:10.7717/peerj.973)
Supplement: Supplemental Information 1 [file peerj-03-973-s001.docx]

**Supplementary Material**
